# Psychiatric diagnoses, trauma, and suicidiality

**DOI:** 10.1186/1744-859X-6-12

**Published:** 2007-04-20

**Authors:** Silje K Floen, Ask Elklit

**Affiliations:** 1 BUP for Nordhordland, Kvassnesvegen 44, N-5914 Isdalstø, Norge; 2 Department of Psychology, University of Aarhus, Jens Chr. Skous Vej 4, DK-8000 Aarhus C, Denmark

## Abstract

**Background:**

This study aimed to examine the associations between psychiatric diagnoses, trauma and suicidiality in psychiatric patients at intake.

**Methods:**

During two months, all consecutive patients (n = 139) in a psychiatric hospital in Western Norway were interviewed (response rate 72%).

**Results:**

Ninety-one percent had been exposed to at least one trauma; 69 percent had been repeatedly exposed to trauma for longer periods of time. Only 7% acquired a PTSD diagnosis. The comorbidity of PTSD and other psychiatric diagnoses were 78%. A number of diagnoses were associated with specific traumas. Sixty-seven percent of the patients reported suicidal thoughts in the month prior to intake; thirty-one percent had attempted suicide in the preceding week. Suicidal ideation, self-harming behaviour, and suicide attempts were associated with specific traumas.

**Conclusion:**

Traumatised patients appear to be under- or misdiagnosed which could have an impact on the efficiency of treatment.

## Background

Based on data from a large nationally representative sample of people participating in the National Comorbidity Survey (NCS) [1], 60% of men and 50% of women reported to have experienced a traumatic event at some point in their lives, with the majority of them having been exposed to two or more traumatic events. The prevalence of trauma exposure among psychiatric populations has been found to be higher than in the rest of the population. Five studies have reported childhood and sexual abuse in between 34% and 81% of patients with severe mental illness (SMI) [2]. In five other studies the exposure to physical and sexual violence varied between 43% and 81% in patients with SMI [2]. In addition, a significant rate (43%) of exposure to car and work accidents has also been reported in SMI patients [3]. A 90% lifetime trauma exposure has been reported among psychiatric patients [4]. Likewise, another study found that 61% of the patients in a psychiatric setting had experienced at least one traumatic event [5]. Thus, psychiatric patients appear to have been more exposed to traumatic events than the general population.

While the NCS [1] found a 5% lifetime prevalence of PTSD among men, and 10% among women in the general population, and 8% and 20% among traumatized men and women, only four studies [3,6-8] have assessed the PTSD prevalence in psychiatric populations, ranging from 29% to 43%. The prevalence ascends to 48% – 66% when one only includes patients who have been exposed to traumatic events. Neither of the abovementioned [3,7,8] found gender differences in their psychiatric populations. Moreover, the likelihood of developing PTSD after traumatic exposure appears to be significantly elevated in patients with SMI. Patients with a lifetime history of PTSD were at 8 and 10 times greater risk for anxiety and psychotic disorders, respectively, than those without such history, and suggested that traumatic experiences and PTSD may have a great impact on the development and course of various other psychiatric disorders [5].

High comorbidity has been found between PTSD and other psychiatric disorders, particularly depressive disorders, anxiety disorders, and substance abuse disorders. The NCS showed that 59% of men and 44% of women with PTSD also met the criteria for three or more other psychiatric diagnosis [1]. The NCS found that 48% of the men and 49% of the women with PTSD also suffered from depression, in particular major depression, and the comorbidity ratio of PTSD and anxiety disorders ranged from 2.4 to 7.1, with social phobia and simple phobia co-occurring most frequently with PTSD (ibid.).

The comorbidity between PTSD and depression may in part be explained by a significant symptom overlap between the two disorders [9]. When the symptoms emerge after a traumatic experience, they are likely to be symptoms of PTSD. Distinguishing the timing of symptoms relative to a traumatic event is crucial, although the task may be complex, i.e. in instances where individuals have experienced multiple traumas or traumatic events in their childhood. The comorbidity may also, in part, be due to sequential causation with depressive disorders occurring secondary to chronic PTSD [9]. The NCS found that in 78% of the subjects with comorbid PTSD and depression, the depressive disorder followed the diagnosis of PTSD [1]. However, a history of depressive disorders predicted [10] the development of PTSD following trauma exposure, indicating that the two disorders are independent, but closely inter-related responses to traumatic experiences. The comorbidity ratio between PTSD and bipolar disorder has been found to be 10.4 in men and 4.5 in women [1]. Indication of childhood mania is a risk factor for both trauma exposure and PTSD, with manic episodes, in particular, increasing the risk for trauma exposure in individuals with the disorder [11].

PTSD and the various anxiety disorders also share overlapping symptoms [9]. The comorbidity between PTSD and panic disorder appears to depend on trauma type [12], i.e. traumatic events that involve unpredictability are more likely to lead to comorbid PTSD and panic attacks, suggesting that these disorders may be interwoven, rather than comorbid. Panic attacks usually start occurring subsequent to the development of chronic PTSD [13].

Two recent factor analytic studies of PTSD symptoms have proposed models that more radically differ from the DSM-IV in that they go beyond modelling avoidance as two separate factors. Simms et al. [14] suggested a four-factor model with re-experiencing, avoidance, dysphoria, and arousal factors. The dysphoria factor was comprised of the emotional numbing symptoms and the irritability/anger, difficulty sleeping, and difficulty concentrating symptoms. The hypervigilance and exaggerated startle response symptoms comprised the arousal factor. The confirmatory factor analyses were based on data from a large sample (N = 3,695) of Gulf War veterans and non-deployed controls using the PTSD Checklist – Military Version (PCL-M; [15]). Simms et al. found this model provided the best fit compared to five other models, and this finding was replicated using different samples. A similar finding is reported in a study of 1116 whiplash sufferers [16]

Until the publication of the analyses by Simms et al. [14] there was a degree of consensus regarding the structure of PTSD symptoms with a four-factor model being widely supported in the research literature: this model separated the traditional avoidance factor into a conscious avoidance and emotional numbing factor [17-20]. However, the findings reported by Simms et al. and Elklit & Shevlin [14,16], suggested a dysphoria factor composed of symptoms from both the avoidance (more specifically emotional numbing) and arousal clusters.

A lifetime comorbidity of 11% between PTSD and schizophrenia or schizophreniform disorder has been found [21]. However, PTSD symptoms are often overlooked in psychotic patients since the treatment objectives usually are treating cognitive disorganization and stabilizing psychoses. Studies have reported psychotic symptoms in 28–40% of PTSD patients [22,23]. PTSD symptoms were found to be more severe in patients who had psychotic symptoms and comorbid major depression [24]. Patients with comorbid psychotic disorder and PTSD showed greater emotional, behavioural, and cognitive disturbance than patients with either of these disorders separately [22].

Data from the NCS suggest that people with PTSD are two to three times more likely to have a lifetime substance use disorder than individuals without it [1] and others have estimated that 30–60% of patients in treatment for substance abuse disorders have lifetime PTSD. A group of women with PTSD comorbid with substance abuse disorder reported more criminal behaviour, fewer outpatient psychiatric treatments, and more suicide attempts than a group of women with PTSD alone [25]. Significantly higher rates of sexual trauma, more borderline personality traits, greater levels of dissociation, and more severe PTSD were found in women with PTSD and comorbid substance abuse disorders than in a comparison group of women with PTSD alone [26]. Moreover, greater use of substances has been found to be associated with elevations in PTSD symptoms and failure to recover from PTSD [27].

Three major causal pathways have been hypothesized to explain the link between substance abuse disorders and PTSD [9]. First, substance use may be a consequence of PTSD providing the individual with temporary relief of painful and uncomfortable symptoms of PTSD. Second, the comorbidity may be a consequence of substance abusers' high-risk lifestyles, which may put them at an additional risk for trauma exposure, including PTSD. Thirdly, individuals with substance abuse disorders may be more prone to developing PTSD following trauma exposure, because of poor coping strategies and changes in brain chemistry that can increase the vulnerability and deteriorate the course of PTSD. Most research evidence appears to support the first hypotheses although the associations appear complex.

Recent research has found that individuals exposed to traumatic events, particularly to childhood physical and sexual abuse, often have dissociative and somatoform symptoms indicating that such symptoms may be trauma-induced phenomena [28]. Several studies of patients with PTSD, dissociative disorders, eating disorders, and borderline personality disorders have found strong associations between pathological dissociation and traumatic experiences (ibid.). Dissociative symptoms correlated more strongly with traumatic experiences in childhood than with borderline psychopathology [29]. As opposed to a comparison group, patients with dissociative disorders reported multifaceted and severe traumatic events, and physical trauma predicted somatoform dissociation whereas sexual trauma predicted somatoform and psychological dissociation [28]. Early onset of chronic, intense, and multifaceted traumatization predicted pathological dissociation.

A number of epidemiological studies support the findings of the NCS that women are more likely to develop PTSD than men (ratio approximately 2:1) although trauma exposure is more common in men [1]. A hypothesized explanation for this gender difference may be due to different characteristics of the traumatic events that the genders are exposed to [30]. Thus, it has been found that men are more frequently exposed to physical assault and combat, whereas women are more frequently exposed to rape and sexual assault [31], and sexual trauma has repeatedly been found to be strongly associated with high rates of PTSD. Other potential explanations have been proposed such as gender-specific attributes of the event, culturally determined roles, culturally determined attribution of guilt, and/or uneven distribution of rights and resources between the genders [32].

PTSD seems to be an important predictor of suicidal behaviour. Ninety-one percent of the young adults who had attempted suicide had at least one psychiatric diagnosis, and that the highest risk for suicidal behaviour was among subjects with PTSD [33]. The likelihood for suicidal attempts among individuals with PTSD was approximately 15 times higher than in individuals without it, and the association between PTSD and suicidal behaviour remained significant after controlling for depressive symptoms [21]. Among suicidal refugees with PTSD, 56% were diagnosed with both PTSD and depressive disorders [34]. In addition, individuals with depression comorbidity reported higher rates of recurrent suicidal thoughts, whereas individuals with PTSD only displayed a higher frequency of suicide attempts.

Self-harming behaviour is deliberate self-injury without the intent to die. A variety of psychiatric diagnoses have been associated with self-harming behaviour [35], i.e. eating disorders, PTSD, substance use disorders, depression, anxiety, schizophrenia, and, in particular, borderline personality disorder. Heightened prevalence of childhood physical and sexual abuse has been found among people with borderline personality disorder [36] and childhood sexual abuse has also been found to be associated with the development of PTSD [37]. Twenty-five percent of incest survivors with PTSD also had self-harming behaviour and suggested that self-harming could be conceptualized as a symptom of PTSD in this population [38]. However, not all incest survivors have self-harming behaviour. Incest survivors with self-harming behaviour score higher on dissociation, depression, and eating disorders than incest survivors without it [39].

The purpose of the present study was (a) to report the occurrences of traumatic events and suicidial behavior and ideation in an acute psychiatric ward in a sample of consecutive patients and (b) to investigate the associations between diagnoses, suicidality and self-harming behaviour. Based on existing research evidence, it was hypothesized that: (1) The prevalence of trauma exposure and PTSD in psychiatric patients would be high; (2) chronic traumatization would be more related to personality disorders than episodic traumatisation, which would be more related to affective disorders such as depression and anxiety; (3) frequent exposure to traumatizing events would be associated with suicidality; and (4) patients who have been exposed to chronic traumatic events in their childhood would be more prone to self-harming behaviour than others.

## Methods

### Participants

All patients arriving in the acute ward of a public psychiatric hospital in Norway during an eight-week period were asked to participate in the current study (N = 139; female = 53%; native Norwegians = 96%; mean age 40 yrs., SD 15.76, median = 38, range 17–86 years). One hundred patients responded (response rate 72%), of which 49% were female and all were native Norwegians. Reasons for non-participation were dementia or mental retardation (15% of non-respondents), inability to answer due to chaotic thoughts and behaviour (39%), insufficient skills in Norwegian (15%), early dismissal from the hospital (15%), and refusal to participate (23%).

Demographic information was acquired for the total sample, including information about gender, age, nationality, and diagnosis. The total sample consisted of individuals with various diagnoses, of which substance use disorders (29%, n = 40), recurrent depression (20%, n = 28), depressive episodes (16%, n = 22), bipolar disorder (12%, n = 16), and paranoid psychosis (10%, n = 14) were most frequent. Other diagnoses included schizophrenia (7%, n = 10), anxiety disorders (7%, n = 10), PTSD (7%, n = 9), borderline personality disorder (6%, n = 8), other personality disorders (9%, n = 12), schizoaffective disorder (4%, n = 6), adjustment disorder (3%, n = 4), and other diagnosis (12%, n = 16). The rate of recidivism was 57% (mean = 2.9; median 1.0; SD = 7.4; range 0–66).

### Measurement

The interview guide consisted of 4 sections. In the first section the participants were asked to indicate whether they had ever been exposed to any of twelve listed traumatic events [1], if such exposure had occurred within the past 12 months, and prior to the past 12 months, and they were asked to indicate whether they had experienced repetitive trauma exposure. The next section included questions about suicidal attempts in the week prior to hospitalization, suicidal thoughts in the past month, and previous suicidal attempts. The third section consisted of questions about self-harming behaviour within the past month, as well as prior to the past month. The fourth section included information from the patient's psychiatric journal about the patient's psychiatric diagnosis, and number of previous psychiatric admissions.

### Procedure

During an eight-week period, the study included all patients arriving in the acute ward of the psychiatric hospital where patients are hospitalized, voluntarily or by force, for a maximum of one week before discharge or transfer to a different ward. Every morning, new arrivals who were stable enough were asked to participate in the study. Some needed to be stabilized before they were asked to participate. The same interviewer interviewed all the patients in their rooms, or in the visiting room when available. They were informed that participation was optional and anonymous, were given a description of the study, and a statement of consent that they were asked to sign. The meaning of the term trauma was explained as "threatening and catastrophic experiences which most people never go through, and which can have serious consequences for a person's life, distinct from other more common negative life events which many people go through, and which often lack the aspect of surprise and sense of loss of integrity". The interview guide was placed in front of the participants so that could follow the questions to be asked. Patients were informed that they could end the interview at any point in time and were given the opportunity to ask questions following the interview.

### Statistical analysis

Pearson correlations and chi-square analyses were used to analyse the data. Analyses were conducted using SPSS-11.

## Results

### Trauma exposure

Ninety-one percent of the participants reported that they had been exposed to at least one traumatic event in their lives, of which 25% reported exposure to a traumatic event in the past 12 months. Repeated exposure to traumatic events over a longer period of time was reported by 69% of the patients. Table 1 shows the percentage of patients that had experienced the individual types of traumatic events. The most frequent traumatic experiences (reported by 40–57% of the patients) were physical assault, childhood neglect, witnessing somebody getting badly injured or killed, and other terrifying experiences outside the normal experiences of most human beings, and the least frequent were combat experience and experiencing natural disaster or fire, reported by 11 and 13% of the patients, respectively. The rate of recidivism was not associated with numbers of traumas, trauma type or PTSD.

**Table 1:** Number of patients who had been exposed to the various traumatic events (n = 100)

Traumatic event	Females	Males	X^2 ^(P)	Percentage (n = 100)	Percentage of total sample
Physical Assault	24	31	ns	55	40
Incest or sexual molestation	19	8	6.76 (.01)	27	19
Rape	16	5	7.86 (.005)	21	15
Combat experience	5	6	ns	11	8
Threatened with a weapon	13	24	4.52 (.05)	37	27
Involved in a life threatening accident	6	15	4.44 (.05)	21	15
Involved in a natural disaster or fire	5	8	ns	13	9
Witnessed someone getting badly injured or killed	17	23	ns	40	28
Seriously neglected as a child	26	18	3.20 (.06)	44	32
Physically abused as a child	15	11	ns	26	19
Suffered a great shock because one of these events happened to someone close	21	15	ns	36	26
Other (terrible experiences that most people never go through)	27	30	ns	57	41

Significantly more women than men had during their lifetime been exposed to incest or sexual molestation, and to rape. Furthermore, significantly more women than men were diagnosed with PTSD (11% versus 2%; χ^2 ^= 5.30, df 1, p < .05) and borderline personality disorder (10% versus 2%; χ^2 ^= 4.33, df 1, p < .05). In contrast, significantly more men than women had been threatened with a weapon and had during their lifetime been involved in a life threatening accident. Also significantly more men than women were diagnosed with substance abuse disorders (39% versus 19%; χ^2 ^= 6.35, df 1, p < .05). No other significant differences were found between the genders in terms of trauma exposure or type of diagnosis.

### Trauma exposure and PTSD

Six of the seven respondents with PTSD (86%) reported that they had been exposed to incest or sexual molestation; four (57%) had been raped; five (71%) had been physically assaulted; five (71%) had witnessed someone being injured or killed; three (43%) had been seriously neglected in childhood; three (43%) had suffered great shock because someone close to them had been exposed to some of the listed traumatic events; two (22%) had been exposed to a natural disaster or fire; two (29%) had been physically abused in their childhood; one (14%) had been threatened with a weapon; one (14%) had been in combat during war; one (14%) had been involved in a life threatening accident; and six (86%) reported having been exposed to other terrifying experiences that most people never experience. It is thus evident that multiple traumatic events were common in the patients diagnosed with PTSD.

### PTSD comorbidity

The comorbidity of PTSD and other psychiatric disorders was high (78%). Only two (22%) of the total number of PTSD patients (N = 9) did not have any comorbid disorders. Two patients had the additional diagnosis of recurrent depression, two of depressive episodes, two of schizo-affective disorder, one patient of borderline personality disorder, two patients of anxiety disorders, and one of substance abuse disorders. Thus, it is evident that PTSD was often comorbid with several additional psychiatric disorders.

### Trauma exposure and other diagnoses

Due to the comorbidity of PTSD with recurrent depression, borderline personality disorder, substance use disorder, depressive episodes, schizoaffective disorder, and anxiety disorders chi-square analyses were performed for the associations between those disorders and trauma type. Recurrent depression was significantly associated with rape (χ^2 ^= 3.75, df 1, p < .05). Borderline personality disorder was significantly associated with incest or molestation (χ^2 ^= 7.54, df 1, p < .01) and childhood physical abuse (χ^2 ^= 8.07, df 1, p < .005). Substance use disorders were significantly associated with having been threatened with a weapon (χ^2 ^= 6.01, df 1, p < .01), and witnessing someone being badly injured or killed (χ^2 ^= 5.67, df 1, p < .02). Depressive episodes were significantly associated with physical assault (χ^2 ^= 3.82, df 1, p < .05), being threatened with a weapon (χ^2 ^= 6.75, df 1, p < .01), and being exposed to natural disasters or fire (χ^2 ^= 9.00, df 1, p < .005).

### Suicidiality reports

Sixty-seven percent (35 men and 32 women) of all patients reported increased suicidal thoughts in the month prior to hospitalization, of which. All the PTSD, the borderline-personality disorder, the anxiety disorders, and the adjustment disorder patients reported suicidal thoughts in the past month. In addition, 31% of the patients (17 men and 14 women) had attempted to commit suicide in the week prior to hospitalization. Sixty-two percent of the respondents (30 men and 32 women) had previously attempted to commit suicide. All the patients with schizoaffective disorder, borderline personality disorder, anxiety disorders, and adjustment disorders belonged to this group (n = 21), as did 86% of the PTSD patients (n = 6). There were no significant gender differences found in terms of suicidal ideation or suicidal behaviour.

### Suicidiality, trauma exposure, and diagnoses

A correlation analysis revealed a significant positive association between suicidal ideation in the previous month and having experienced incest or sexual molestation (χ^2 ^= 5.38, df 1, p < .02), having witnessed someone being seriously injured or killed (χ^2 ^= 5.10, df 1, p < .05), and having had other terrifying experiences that most people never have to go through (χ^2 ^= 11.26, df 1, p < .001). Moreover, there was a significant association found between previous suicide attempts and rape (χ^2 ^= 4.05, df 1, p < .05), and previous suicide attempts and being threatened with a weapon (χ^2 ^= 4.66, df 1, p < .05). Chi-square analyses were performed in order to investigate in more detail the relationship between the different diagnoses and suicidality. However, increased suicidal thoughts in the month prior to hospitalization were significantly associated with PTSD (χ^2 ^= 3.71, df 1, p < .05), bipolar disorder (χ^2 ^= 8.82, df 1, p < .005), borderline personality disorder (χ^2 ^= 3.71, df 1, p < .05), and anxiety disorders. Previous suicide attempts were found to be significantly associated with schizoaffective disorder (χ^2 ^= 3.23, df 1, p < .07), borderline personality disorder (χ^2 ^= 4.61, df 1, p < .05), and anxiety disorders (χ^2 ^= 6.06, df 1, p < .01), but not with PTSD.

### Self-harm, trauma, and diagnoses

Seventeen percent of the respondents had had self-harming behaviour in the month prior to hospitalization, of which 47% were men (n = 8), and 53% were women (n = 9). Thirty percent had had self-harming behaviour earlier in life (47% men; n = 14 and 53% women; n = 16). The difference between the genders was non-significant in both instances. The only significant correlation found between exposure to trauma type and self-harming behaviour was found between having been raped and self-harming behaviour earlier in life (χ^2 ^= 6.34, df 1, p < .05). However, a chi-square analysis of the associations between the diagnosis and self-harming behaviour revealed a significant association between PTSD and self-harming behaviour earlier in life (χ^2 ^= 6.15, df 1, p < .01). Borderline personality disorder was also associated with self-harming behaviour earlier in life (χ^2 ^= 11.13, df 1, p < .005), as were anxiety disorders (χ^2 ^= 6.33, df 1, p < .01). The only significant association between suicidality and self-harming behaviours was found between earlier suicidal attempts and self-harming behaviours earlier in life (r = .42, p < .01).

## Discussion

Early longitudinal studies suggested that stressful experiences were equally determined by psychiatric disorders, as they were determinants of such disorders [40]. The main finding of the present study was the extremely high lifetime prevalence of trauma exposure in the sample of psychiatric patients (91%) and the relatively high prevalence of recent trauma exposure (25% in the past year), which was in accordance with the first hypothesis, and which supports the existing evidence of an association between traumatic experiences and the development of psychiatric illnesses.

However, as has been noted, distinguishing between cause and effect in this connection may be difficult, as psychiatric illnesses may predispose people to being exposed to traumatic events, and traumatic events may in turn increase psychiatric symptoms. Nonetheless, and despite the high prevalence of trauma exposure found in the sample, only 7% of the respondents had the diagnosis of PTSD, which is in sharp contrast with the previously reported PTSD prevalence in treatment seeking populations in excess of 50% [1]. High comorbidity has previously been found between PTSD and a large number of other psychiatric illnesses, and this was also found here (7 of the 9 patients in the population had comorbid diagnoses). While the PTSD sample was small in the present study, six different comorbid diagnoses were reported.

A closer look at the traumatic experiences reported by the PTSD subjects revealed a large number of traumatic situations that the subjects had been exposed to, leaving no doubt about their traumatization. However, the evidence also indicates that traumatisation was frequent in many of the other diagnostic groups. This may potentially be taken as an indication for the need for repetitive trauma exposure before a person develops diagnosable PTSD, or alternatively, as an indication that PTSD was under-diagnosed in the sample. Avoidance symptoms are an important aspect of PTSD and may lead to false negatives in diagnosing patients with PTSD, as they may avoid talking about their traumatic experiences in an effort to avoid the pain associated with memories of the traumatic event. As a result, symptoms of PTSD may be camouflaged by symptoms of comorbid illnesses, such as depression, anxiety, substance use disorder etc. In addition, the overlap of symptoms may make it difficult to distinguish between the different diagnoses, and the trauma history of the individual may not be adequately investigated within clinical settings.

However, as has been noted, research evidence appears to indicate that traumatization may be the cause for certain other diagnoses, such as when a person starts abusing alcohol or other substances in an effort to alleviate the pain associated with the traumatic experience and the symptoms associated with it, and when a person becomes depressed, potentially because of symptoms of PTSD, such as social isolation due to avoidance of people, places, and other stimuli associated with a traumatic experience.

The research evidence did not find clear support for hypothesis no. 2 which stated in accordance with Terr [41] that single event trauma exposure would be associated with axis I disorders, such as depression and anxiety, whereas repetitive trauma would be associated with axis II disorders, i.e. personality disorders. PTSD, anxiety, and borderline personality disorder were all associated with incest or sexual molestation. The latter was moreover associated with childhood physical abuse and being threatened with a weapon, whereas PTSD and recurrent depression were associated with rape. Depressive episodes were associated with physical assault, being threatened with a weapon, and exposure to natural disaster or fire, and substance use disorders were associated with witnessing injury or death, and being threatened with a weapon. Finally, schizophrenia was found to be associated with witnessing injury or death. Thus, the evidence indicated that trauma is an important component of various psychiatric disorders and provides further evidence that PTSD may be under-diagnosed in this population.

Hypotheses nos. 3 and 4 stated that there would be an association between suicidal ideation/suicidal behaviour and traumatization, as well as between self-harming behaviour and traumatization. The results showed that a large percentage of patients with adjustment disorders, PTSD, and borderline personality disorders had attempted suicide in the week prior to hospitalization, but potentially due to the small size of these diagnostic groups these associations were non-significant, making it impossible to draw conclusions based on this evidence. In fact, no significant associations were found between suicidal attempts in the past week and diagnostic group or trauma type.

However, exposure to incest or sexual molestation, witnessing serious injury or death, and other terrifying experiences not listed were associated with increased suicidal thoughts in the previous month, whereas having been raped and threatened with a weapon were associated with previous suicidal attempts. Exposure to these experiences, with the exception of being threatened with a weapon, was common in the PTSD sample: incest or sexual molestation (6 of 7), witnessing serious injury or death (5 of 7), other terrifying experiences (6 of 7), and rape (4 of 7).

Moreover, increased suicidal thoughts in the month prior to hospitalization were associated with PTSD, bipolar disorder, borderline personality disorder and anxiety disorders, while only borderline personality disorders, anxiety disorders, and schizoaffective disorders were associated with prior suicidal attempts. It is likely that the association between trauma exposure on the one hand, and suicidality and suicidal ideation on the other, is stronger than these data indicate, as the small sample size decreases the significance of the findings.

Furthermore, there was a significant association between previous self-harming behaviour and the diagnoses of PTSD, borderline personality, and anxiety disorders, whereas self-harming behaviour in the month prior to hospitalization was associated with substance use disorders. However, the only trauma type that was significantly associated with previous self-harming behaviour was rape, which was also associated with previous suicide attempts. Four of the seven patients with PTSD, 3 of the 7 patients with BPD, and 4 of the 9 patients with anxiety disorders reported that they had been raped, indicating an association between traumatization, self-harming behaviour, and suicidality, although the small size of the present sample may undermine this association. Thus again, the evidence appears to support the assertion that PTSD may be under-diagnosed in this sample, although the small sample size reduces the value of any conclusions drawn based on these findings.

The generalisability of the results of the study is limited by the region of the data collection and by the number of participants. If the inquiry about trauma and suicidal behaviour was integrated in the diagnostic process, a more adequate description of the relationship between trauma and diagnoses might emerge. The way of inquiry is similar to a procedure that initiated a self-report survey after the intake interview [42]. Nearly all the patients abuse histories were consistent with later reports obtained in the survey and the latter revealed twice as many as the intake reports. Criticism may also be raised concerning the ability of SMI patients to report traumatic events reliably. Evidence exists that trauma history and PTSD assessment can yield reliable information in patients with SMI [43].

## Conclusion

While many patients with serious and persistent psychiatric disorders have experienced trauma, this is rarely reflected in the diagnoses, and thus, is not included in the treatment [44]. The results of the current study appear to provide support for this view, as it is evident that this patient group included highly traumatized individuals and despite that only 7% of the respondents had the diagnosis of PTSD. Thus, based on previous findings it is suggested that the low prevalence of PTSD may reflect a tendency to neglect inquiring about the patient's trauma history during the assessment phase in psychiatric settings, and that this may have serious implications, leading to prolonged and ineffective treatment (ibid.) and, potentially, the development of secondary diagnoses, such as depression, panic attacks, and substance use disorders, to name a few. In addition, the recognition and validation of problems associated with trauma is often stated by patients as central to their experiences of their disorders [45].
